# Plasticity in the *Drosophila* larval visual system

**DOI:** 10.3389/fncel.2013.00105

**Published:** 2013-07-04

**Authors:** Abud J. Farca-Luna, Simon G. Sprecher

**Affiliations:** Institute of Cell and Developmental Biology, Department of Biology, University of FribourgFribourg, Switzerland

**Keywords:** *Drosophila*, larval visual system, sensory systems, neuronal circuits, cAMP pathway

## Abstract

The remarkable ability of the nervous system to modify its structure and function is mostly experience and activity modulated. The molecular basis of neuronal plasticity has been studied in higher behavioral processes, such as learning and memory formation. However, neuronal plasticity is not restricted to higher brain functions and it may provide a basic feature of adaptation of all neural circuits. The fruit fly *Drosophila melanogaster* provides a powerful genetic model to gain insight into the molecular basis of nervous system development and function. The nervous system of the larvae is again a magnitude simpler than its adult counter part, allowing the genetic assessment of a number of individual genetically identifiable neurons. We review here recent progress on the genetic basis of neuronal plasticity in developing and functioning neural circuits focusing on the simple visual system of the *Drosophila* larva.

## INTRODUCTION

The nervous system displays the remarkable ability to change in response to a continuously altering environment. In such a manner, internal and external stimuli remodel neuronal anatomy and function. This adaptive and dynamic process is known as neuronal plasticity. Neuronal adjustments make use of different cellular and molecular mechanisms. At the genetic level, differential gene expression can lead to changes in protein synthesis related to cytoskeletal and synaptic structure. Neuronal plasticity can be reflected by functional changes in ionic transport and electrical activity, a process known as functional plasticity. Structural modifications may result in neuroanatomical changes including circuitry remodeling. Therefore, understanding mechanisms underlying neuronal plasticity provides important insights into global brain function. Here, we review recent progress concerning neuronal plasticity in a readable nervous system, the larval brain of the fruit fly *Drosophila melanogaster*.

Modifications of synaptic strength and structure provide basic mechanisms underlying the formation of memories (Kandel, 2001). The cyclic adenosine monophosphate pathway (cAMP) has been linked to this form of plasticity. This pathway is required for learning in diverse animal species suggesting a high degree of evolutionary conservation. Studies in adult flies involving olfactory learning protocols have been instrumental to identify genes implicated in this process. Biochemical analyses have identified many genes coding for proteins involved in the cAMP signal pathway. Furthermore, many of the genes identified in the fly have homologous genes with similar function in other species (Zhong and Wu, 1991; Dubnau and Tully, 1998; Kandel, 2001; Ho et al., 2011).

The *Drosophila* larva is simple in comparison to its adult counterpart, containing only about one-tenth of neurons in its nervous system. However, the fly larva is able to perform complex and simple stereotypical behaviors. A conspicuous innate behavior of *Drosophila* larva is its aversion to light (Sawin-McCormack et al., 1995; Keene et al., 2011). An intriguing behavioral switch of this negative photobehavior occurs shortly before the larva undergoes metamorphosis to pupal stage and become photo-neutral. The mechanisms behind the switch are undetermined but very likely behavioral modifications result from plasticity in the underlying neuronal circuit.

The cAMP pathway is required for experience dependent neuronal plasticity in the visual system, a form of plasticity that does not depend on reinforcement by training in contrast to associative learning. A common molecular pathway drives both structural and functional plasticity during development in a set of four neurons, termed “lateral neurons,” LNs in the *Drosophila* larva. These LNs are involved in processing visual information and the control of circadian rhythmic activity (Malpel et al., 2002). Exposure of *Drosophila* larva to different light and darkness combinations confers compensatory plasticity to the neurons explored (Yuan et al., 2011). The discovery opens the repertoire of new actions of the cAMP pathway in neuronal plasticity, supporting the idea that cAMP could be required for different kinds of synaptic plasticity at different phases during the life of an animal (Zhong and Wu, 2004). Additionally, it has been reported that dendritic development of serotonergic neurons in the same visual circuit involves Rac for light-dependent plasticity (Rodriguez Moncalvo and Campos, 2009). We put here these recent discoveries of light-dependent plasticity in the context of current knowledge of the mechanisms regulating neuronal plasticity.

## SHEDDING LIGHT ON THE LARVAL EYE

**FIGURE 1:**
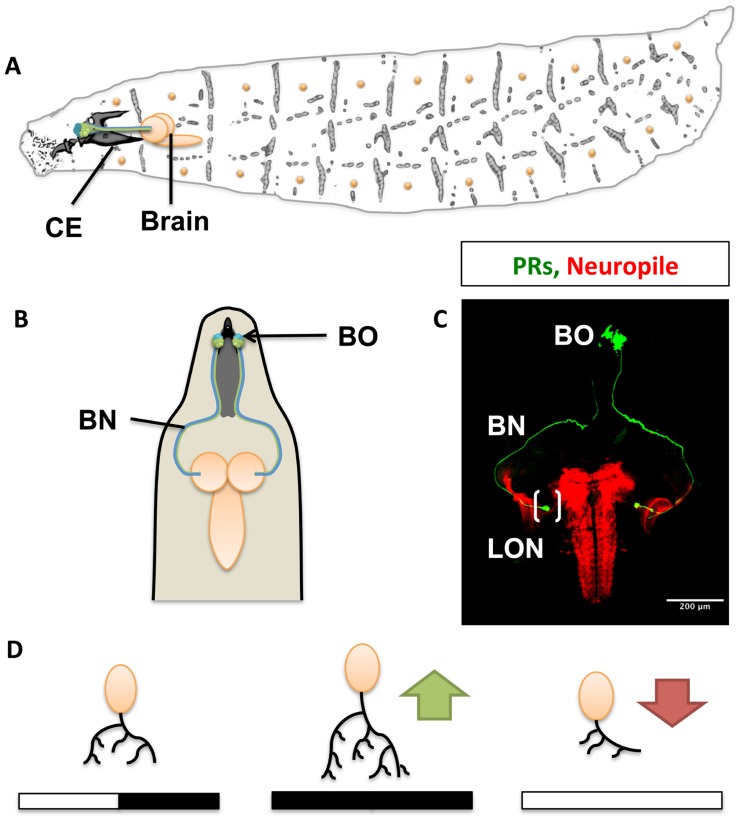
**The visual system of the *Drosophila* larva.** **(A)** Complete overview of *Drosophila* larva showing the localization of the larval eye and projection to the central brain. CE, cephalopharyngeal skeleton. **(B)** Schematic representation showing the larval eye and projection to both hemispheres of the central brain. **(C)** Confocal scanning of the Bolwig organ (BO) and Bolwig nerve (BN, in green) targeting the larval optic neuropile (LON) shown in brackets, the brain neuropiles are shown in red. **(D)** Light exposure effects on dendritic arbor length and activity. Constant darkness leads to increased arbor length and activity (green arrow) in contrast to exposure to 12 h of light and 12 h of darkness. Light hyperstimulation reduces dendritic arbor length and activity (red arrow) in a compensatory manner to high stimulation.

Light is a vital environmental cue for living organisms. *Drosophila* larvae sense light through a group of 12 photoreceptors (PRs) of the larval eye (also termed Bolwig organ, BO). The larval eye is situated anteriorly and lateral, at the tip of the larva, associated with the cephalopharyngeal skeleton (Steller et al., 1987; Green et al., 1993; **Figures 1A,B**). Four PRs express exclusively *rhodopsin5 *(*rh5*) and detect blue light, the remaining eight express only *rhodopsin6* (*rh6*) detecting green light (Malpel et al., 2002; Sprecher et al., 2007; Sprecher and Desplan, 2008). Light perceived by the eye has at least two important behavioral functions: rapid phototaxis and control of circadian rhythms. Larvae react with a strong and stereotypic avoidance response to light exposure. This negative phototaxis or light avoidance requires functional PRs in the larval eye. Interestingly, only the blue-sensitive Rh5-PRs are required for light avoidance, while the Rh6-PRs do not appear to contribute to visual navigation. A second function of the larval eye is the light-dependent entrainment of the molecular clock thereby controlling circadian rhythmic activity. For entrainment of the molecular clock either PR subtype is sufficient, showing divergent and overlapping functions of larval PRs. Larval PRs form a neurite bundle (the Bolwig nerve, BN) projecting jointly to a small neuropil compartment termed larval optic neuropil (LON), where they contact target neurons (**Figure 1C**; Mukhopadhyay and Campos, 1995; Malpel et al., 2002; Sprecher et al., 2011). 

## DEVELOPMENTAL FUNCTIONAL PLASTICITY OF LARVAL PRs

Both larval PR subtypes develop during embryogenesis and are therefore fully differentiated and functioning during larval stages containing the neurotransmitter acetylcholine. Interestingly, during pupation, a part of the larval eye is maintained into adulthood giving rise to the “Hofbauer–Buchner” eyelet (HB-eyelet or eyelet), a small extraretinal light-sensing organ required for some aspects of circadian rhythm control (Helfrich-Forster et al., 2002; Veleri et al., 2007). During the transformation, the larval Rh5-PRs undergo a unique form of sensory plasticity switching the expression of their sensory receptor gene from Rh5 in the larva to Rh6 in the adult eyelet. This switch of sensory receptor genes is controlled by the steroid hormone Ecdysone (Sprecher and Desplan, 2008). A similar change of *rhodopsin* expression was also reported in pacific pink salmon and rainbow trout. Freshly hatched fishes express a UV-sensitive *rhodopsin*, which during later stages changes to blue-sensitive *rhodopsins*. In both cases the switch of *rhodopsins* may be associated with the change in life style: mature fish migrate to deeper areas, where UV light is less abundant (Cheng and Flamarique, 2004, 2007; Cheng et al., 2006). Similarly, the eyelet is located below retina and head capsule where light with longer wavelength is likely more abundant and thus perceived rather by Rh6 than Rh5. The transformation of the larval eye into the adult eyelet is associated with a profound restructuring of the axonal projection and termini from the larval to the adult stage. The transformation also includes a change of neurotransmitter from larva to the adult. During larval stages PRs are exclusively cholinergic (Keene et al., 2011), while in the adult eyelet-PRs are both cholinergic and histaminergic (Veleri et al., 2007). Thus, during metamorphosis a number of distinct forms of plasticity occur in the visual system controlled by the systemically acting Ecdysone receptor pathway.

## STRUCTURAL PLASTICITY IN THE *Drosophila* LARVAL VISUAL CIRCUIT

While the cAMP pathway has been characterized in mediating olfactory associative learning in the adult fly, it has recently been implemented in controlling structural and functional activity dependent plasticity in LNs. Hyperstimulation by constant light exposure, normal light conditions and light-deprivation results in changes in dendritic arbor length of LNs (**Figure 1D**). Increased light exposure results in a shortage of individual dendritic length and developmental delay of the third instar larval stage (L3). On the contrary, under constant darkness (DD) the length of dendritic arbors of individual LNs increases (Yuan et al., 2011). Stimulating the larval eye in an intact brain preparation with wavelengths of 488 or 543 to activate either Rh5 or Rh6 elicited calcium responses in LN axon terminals. These responses were dependent on laser intensity and duration. The calcium response was diminished in larvae maintained under light hyperstimulation, while it was increased in larvae reared under constant darkness. The functional results show a compensatory effect of the dendritic length to different light protocols. Similarly, synaptic activation of PRs resulted in a reduction of LN dendrites resembling the effects of constant light, while PR inactivation lead to dendritic growth of the LN dendrites. This suggests that communication between PRs and LN is crucial for dendrite maintenance. Interestingly, most genes impacting on dendritic length have also been reported as modulators of plasticity during learning and memory formation. Similarly, structural plasticity inhibitory effects occurred after manipulating downstream genes of the cAMP pathway. Mutations or knockdowns of these genes prevented structural and functional effects, and maintained the responsiveness of the LNs to different light dark stimulation uniform (Yuan et al., 2011). Thus, second order neurons in the visual system underlie large structural plasticity in their dendritic arbors, while remaining fully functional, suggesting widely occurring compensatory and homeostatic mechanisms.

## BEHAVIORAL PLASTICITY DURING LARVAL LIFE

The third instar larval stage is divided in two sub-stages, the foraging and the wandering phase. While foraging third instar larvae display generally similar behaviors like second or first instar animals, during the wandering stage the animal typically leaves the food source and moves to a proper pupariation site. Interestingly the change from foraging to wandering third instar is also accompanied with a change of photobehavior. During most of its life the larva displays a strong stereotypic negative behavior, this response disappears during wandering larvae when the animals are typically photo-neutral (Sawin-McCormack et al., 1995; Mazzoni et al., 2005). While the neuronal basis of this behavioral plasticity is not completely understood two pairs of neurons have been shown to be required in maintaining larvae photophobic (Gong et al., 2010). These neurons are referred to “NP394”-neurons since the NP394-Gal4 line marks exclusively these four neurons. Genetic inhibition of NP394-neurons switches photophobic behavior at all larval stages, suggesting a possible mechanism modifying photobehavior during late larval stages. Similarly, a pair of serotonergic neurons has been shown to specifically innervate the LON during late larval stages. Formation of serotonergic synapses requires the presence of the larval Rh6-PR subtype, since permanent selective genetic ablation of Rh6-PRs results in a lack of serotonergic innervation, while Rh5-PRs ablation has no effect. Neurite outgrowth further requires activity of the transmembrane protein Rac for light-dependent synaptic plasticity (Rodriguez Moncalvo and Campos, 2005).

Moreover, larvae are able to form associative memories, a process requiring cAMP dependent synaptic plasticity in the adult. Since light is a strong aversive stimulus, it can be used as a negative reinforcement for olfactory cues (von Essen et al., 2011). Thereby light functions as unconditioned stimulus altering the preference for olfactory stimuli. Conversely, the preference to light can be changed when either rewarded with sugar as positive reinforce or when light preference is punished by salt or electric shock (von Essen et al., 2011). The current knowledge suggests that experience dependent plasticity and associative memories require the cAMP pathway. Whether plastic changes in the central nervous system of the larva are similar to those of adult after olfactory learning occurs has to be still elucidated.

## CONCLUDING REMARKS

Neuronal plasticity is a prominent process during development, environmental experience and learning. The cAMP pathway seems to be a common feature controlling the neuroplastic process, but how the cAMP pathway is controlled in distinct contexts is still an open question. A first approach is to investigate the role of developmental hormones, ions and neurotransmitters and transmembrane proteins for the activation of the specific pathway for neuronal plasticity. Recent studies at the *Drosophila* NMJ (neuromuscular junction) have linked ion channels and signaling transducers with the cAMP pathway and its role in synaptic plasticity (Zhong and Wu, 2004; Lee and Wu, 2010; Tsai et al., 2012a, b). In the central nervous system, at least three genes have been identified to also contribute with the process: *Rac* (Rodriguez Moncalvo and Campos, 2009), *Notch* (Wang et al., 2004; Seugnet et al., 2011), and *babos*-1 (Yuan et al., 2011). However, their mechanisms of action in experience dependent plasticity have to be explored. On the other hand, the activation of the cAMP pathway leads to functional and structural changes, very likely involving *de novo* synthesis or degradation of cytoskeleton and synaptic proteins. Experience dependent plasticity in invertebrate models such as the fruit fly is likely to provide deep insights and novel mechanisms of how the environment models the nervous system.

## Conflict of Interest Statement

The authors declare that the research was conducted in the absence of any commercial or financial relationships that could be construed as a potential conflict of interest.
